# Biomechanical evaluation of a novel biomimetic artificial intervertebral disc in canine cervical cadaveric spines

**DOI:** 10.1002/jsp2.1251

**Published:** 2023-02-21

**Authors:** Celien A. M. Jacobs, Remco J. P. Doodkorte, S. Amir Kamali, Abdelrahman M. Abdelgawad, Samaneh Ghazanfari, Stefan Jockenhoevel, J. J. Chris Arts, Marianna A. Tryfonidou, Björn P. Meij, Keita Ito

**Affiliations:** ^1^ Orthopedic Biomechanics, Department of Biomedical Engineering Eindhoven University of Technology Eindhoven Noord‐Brabant The Netherlands; ^2^ Department of Orthopedic Surgery, Research School CAPHRI Maastricht University Medical Center Maastricht Limburg The Netherlands; ^3^ Department of Clinical Sciences, Faculty of Veterinary Medicine Utrecht University Utrecht Utrecht The Netherlands; ^4^ Aachen‐Maastricht Institute for Biobased Materials, Faculty of Science and Engineering Maastricht University Geleen Limburg The Netherlands; ^5^ Department of Biohybrid and Medical Textiles (BioTex), AME – Institute of Applied Medical Engineering Helmholtz Institute, RWTH Aachen University Aachen Nordrhein‐Westfalen Germany

**Keywords:** biomechanical, biomimetic artificial disc, cervical spine, kinematics, total disc replacement

## Abstract

**Background Context:**

Cervical disc replacement (CDR) aims to restore motion of the treated level to reduce the risk of adjacent segment disease (ASD) compared with spinal fusion. However, first‐generation articulating devices are unable to mimic the complex deformation kinematics of a natural disc. Thus, a biomimetic artificial intervertebral CDR (bioAID), containing a hydroxyethylmethacrylate (HEMA)—sodium methacrylate (NaMA) hydrogel core representing the nucleus pulposus, an ultra‐high‐molecular‐weight‐polyethylene fiber jacket as annulus fibrosus, and titanium endplates with pins for primary mechanical fixation, was developed.

**Purpose:**

To assess the initial biomechanical effect of the bioAID on the kinematic behavior of the canine spine, an ex vivo biomechanical study in 6‐degrees‐of‐freedom was performed.

**Study Design:**

A canine cadaveric biomechanical study.

**Methods:**

Six cadaveric canine specimens (C3‐C6) were tested in flexion‐extension (FE), lateral bending (LB) axial rotation (AR) using a spine tester in three conditions: intact, after C4‐C5 disc replacement with bioAID, and after C4‐C5 interbody fusion. A hybrid protocol was used where first the intact spines were subjected to a pure moment of ±1 Nm, whereafter the treated spines were subjected to the full range of motion (ROM) of the intact condition. 3D segmental motions at all levels were measured while recording the reaction torsion. Biomechanical parameters studied included ROM, neutral zone (NZ), and intradiscal pressure (IDP) at the adjacent cranial level (C3‐C4).

**Results:**

The bioAID retained the sigmoid shape of the moment‐rotation curves with a NZ similar to the intact condition in LB and FE. Additionally, the normalized ROMs at the bioAID‐treated level were statistically equivalent to intact during FE and AR while slightly decreased in LB. At the two adjacent levels, ROMs showed similar values for the intact compared to the bioAID for FE and AR and an increase in LB. In contrast, levels adjacent to the fused segment showed an increased motion in FE and LB as compensation for the loss of motion at the treated level. The IDP at the adjacent C3‐C4 level after implantation of bioAID was close to intact values. After fusion, increased IDP was found compared with intact but did not reach statistical significance.

**Conclusion:**

This study indicates that the bioAID can mimic the kinematic behavior of the replaced intervertebral disc and preserves that for the adjacent levels better than fusion. As a result, CDR using the novel bioAID is a promising alternative treatment for replacing severely degenerated intervertebral discs.

## Graphical abstract

This study assessed the initial biomechanical effect of the bioAID on the kinematic behavior of the canine spine, an ex vivo biomechanical study in 6‐degrees‐of‐freedom was performed. The results that the bioAID can mimic the kinematic behavior of the replaced intervertebral disc and preserves that for the adjacent levels better than fusion.

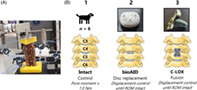

## 1 INTRODUCTION

Currently, the golden standard to treat severely degenerated intervertebral discs (IVDs) is anterior cervical discectomy and fusion (ACDF). ACDF has shown promising clinical results, but several limitations remain to fuse the vertebrae.^1^, ^2^, ^3^, ^4^, ^5^, ^6^, ^7^ It is hypothesized that adjacent segments need to compensate for the altered loading pattern due to the loss of motion at the index level. Research has shown that 92% of patients showed radiographic degeneration of the adjacent segments 5 years post fusion surgery.^8^ Other studies have reported different rates for the incidence of symptomatic adjacent segment disease (ASD). One study found the prevalence of symptomatic ASD in 6.2% of the cases after single level ACDF at different follow‐up periods ranging between 5 and 15 years.^9^ At 5 years follow‐up, the rate of ASD after ACDF was found to be 10.9%.^10^ On the other hand, Wu et al. (2019) only found 2.9% of the patients that needed a second surgery to treat ASD at 16 years follow‐up.^11^ As a result, cervical disc replacement (CDR) has been proposed as an alternative treatment that aims to restore motion of the treated spinal level to reduce the risk of adjacent segment pathology compared with fusion. Xie et al. (2016) compared data of 20 randomized controlled trials with a total of 4004 patients with a follow‐up of 2 years; results indeed showed that CDR was statistically superior to ACDF in the development of adjacent segment disease (ASD) with a risk ratio of 0.62 and a 95% confidence interval (0.43, 0.88).^1^ This conclusion is supported by Wu et al. (2017) who also reported fewer rates of ASD in the CDR group compared to ACDF, although, according to the authors, based on relatively low‐quality evidence.^5^ Besides ASD, other clinical outcomes such as arm and neck pain, and patient satisfaction have also shown to be more favorable for CDR compared to ACDF.^1^, ^3^, ^12^, ^13^

**FIGURE 1:**
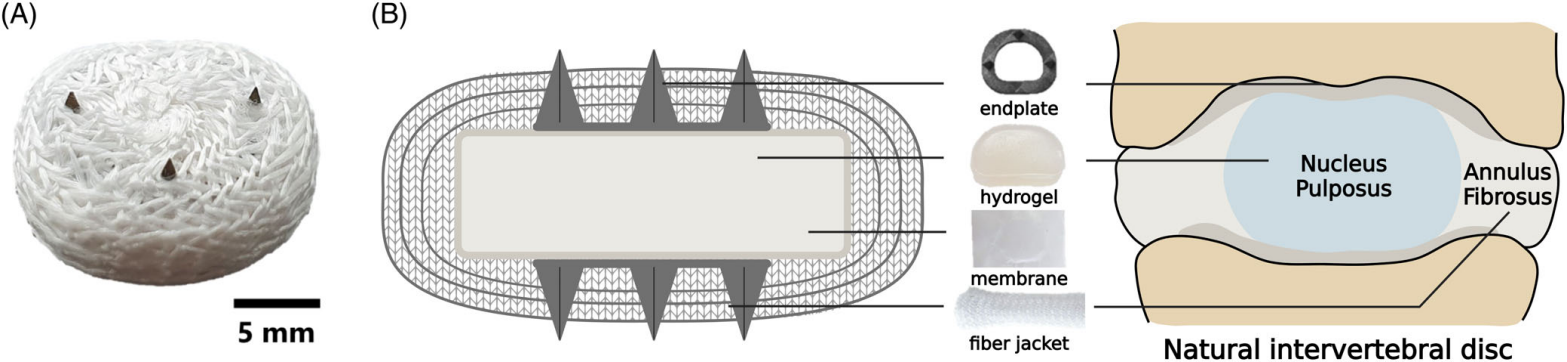
(A) biomimetic artificial intervertebral disc (bioAID). (B) schematic representation of the design of the bioAID and its biomimicry compared to a natural disc.

Despite these promising outcomes, first‐generation articulating ball‐and‐socket disc replacements cannot mimic the complex deformational kinematics of natural IVDs.^14^, ^15^, ^16^ The design of these first‐generation prostheses is often derived from large synovial joint arthroplasties and thus is mainly based on sliding motions, whereas the natural IVD allows motion based on deformation.^17^, ^18^ Previous research has shown that, a first‐generation ball‐and‐socket implant could not reproduce the kinematic signature of an intact spinal segment, unlike a second‐generation with a deformable viscoelastic component.^18^ Another advantage of these second‐generation devices is that these devices have a variable center of rotation (COR), therefore being less susceptible to correct positioning.^19^, ^20^, ^21^ Although these second‐generation devices are already an improvement when compared to first‐generation devices, none of the currently available implants can mimic the osmotic swelling pressure known to be crucial for the biomechanical properties of the IVD tissue, needed to provide its compressive resistance.^14^, ^17^ To better replicate the biomechanical properties of the natural IVD, a biomimetic artificial IVD (bioAID) was developed.^22^, ^23^ This novel prosthesis mimics a number of aspects of the native structure of the IVD and aims to mimic its biomechanical properties. The bioAID design contains a hydrogel core wrapped in a membrane, representing the contained gelatinous swelling nucleus pulposus, a stiff ultra‐high‐molecular‐weight‐polyethylene (UHMWPE) fiber jacket mimicking the tensile load‐bearing of the annulus fibrosus, and a titanium endplate with pins to prevent initial device migration (Figure 1).^22^, ^23^, ^24^ The combination of the hydrogel wrapped with fiber jacket aims to imitate the properties of a natural IVD, like nonlinear viscoelastic behavior, osmotic pressure resulting in prestress of fibers, creep, relaxation, and intradiscal pressure (IDP). Furthermore, it offers stability and shock absorbance while allowing semi‐constrained motion based on deformation.^22^

Since the primary rationale of CDR is to preserve and restore the natural range of motion (ROM), it is of great importance to assess the bioAID's capability to restore the physiological kinematics of the spine. It is hypothesized that the biomimetic structure of the bioAID can maintain normal kinematics at the treated and adjacent levels, thereby minimizing the risk of adjacent segment pathologies in the long term. Therefore, this ex vivo biomechanical study in 6‐degrees‐of‐freedom (6‐DOF) was performed to assess the initial biomechanical effect of the bioAID on the kinematic behavior of the treated and adjacent canine cervical spine segments.

## 2 MATERIALS AND METHODS

### 2.1 Device design

The first prototypes were developed for the lumbar spine, while clinical need, market size, interest of industry, and clinicians showed more feasibility for the cervical spine.^22^, ^23^ As a consequence, the cervical bioAID prosthesis (21 × 14.5 × 5 mm) consisting of an ionized hydrogel surrounded by a membrane and three layers of fiber jacket was re‐sized and re‐designed (Figure 1). This was again resized for a canine model as cervical disc degeneration is also problematic in dogs and the size of the cervical vertebrae is not too different from small humans. Moreover, a canine model will also be used later for in vivo proof‐of‐concept studies. Canine dimensions (14.5 × 13.5 × 4.5 mm) were determined based on CT scans of mixed breed dogs. The hydrogel was prepared by dissolving its components in ultra‐pure water (Table 1). Next, a disc of polyurethane foam (diameter 10 cm × height 0.2 cm, MCF.03, Corpura B.V., Etten‐Leur, The Netherlands) was soaked with the hydrogel solution and polymerized under UV light (UVP XX15L, 365 nm, Analytik Jena, Upland, CA USA) for 2 h. It was subsequently heated to 45°C for 14 h to complete polymerization. After polymerization, the hydrogel core (14 × 13 × 2 mm) was punched out. This hydrogel was sealed (thermal cutter, HSG‐0, HSGM, Walluf, Germany) into an UHMWPE pouch (38 μm thick, 5 g/m^2^, 0.9 μm pore, membrane, DSM Biomedical, Geleen, the Netherlands) to contain the hydrogel. A tube was warp‐knitted (2 × 1 lapping, 8 stitch/cm, Centexbel, Grâce‐Hollogne, Belgium) from multifilament UHMWPE yarn (Dyneema Purity® SGX, dtex110, TS 100, DSM Biomedical, Geleen, Netherlands). The core was then enclosed in three layers of this tubing and manually sewn closed with Dyneema purity® yarn to form an outer jacket. Before closure, a wire‐eroded titanium endplate ring (9 × 8 × 0.3 mm) with 2 mm pins was placed above the innermost layer of the jacket, such that the pins protruded out of the jacket. Prior to implantation in the cadaveric spines, the bioAIDs were swollen under a 50 N load in PBS (Dulbecco's Phosphate Buffered Saline, Sigma Aldrich) for 7 days to reach swelling equilibrium and mimic the compressive load of a natural spine due to the weight of the head.^25^

**TABLE 1:** Chemical components of the HEMA‐NaMA hydrogel solution.

Components of the monomer solution	Function	Mol ratio	Weight (g)
Distilled water	Solvent	0.80	35.74
Sodium methacrylate 99% (NaMA)	Monomer	0.02	5.09
2‐hydroxyethyl methacrylate 97% (HEMA)	Monomer	0.18	55.2
Poly (ethylene glycol) dimethacrylate, average molecular weight 550 nM	Cross‐linker	0.00001	5.75
2,2′ azobis (2‐methylpropionamidine) dihydrochloride, 97%	Initiator	0.0001	0.054

### 2.2 Specimen preparation

Six fresh‐frozen cadaveric cervical canine spines were obtained from donated animals of the Faculty of Veterinary Medicine, Utrecht University, The Netherlands that became available from unrelated experiments. The cadaveric cervical canine spines were thawed at room temperature, and all paraspinal musculature was removed while preserving the IVDs, facet joints, and ligaments. Radiographical screening was performed to exclude specimens with any spinal pathology. Thereafter, the spinal columns were wrapped in PBS‐soaked gauzes and stored overnight in the fridge. Two standard woodscrews were drilled in the cranial (C3) and caudal (C6) endplate to improve the embedding fixation. Next, the spine was vertically aligned using a line laser before embedding it in polymethylmethacrylate resin (Technovit 3040, Heraeus Kulzer GmbH, Wehrheim, Germany). During the experiment, the specimens were kept hydrated by applying PBS.

### 2.3 Biomechanical testing

**FIGURE 2:**
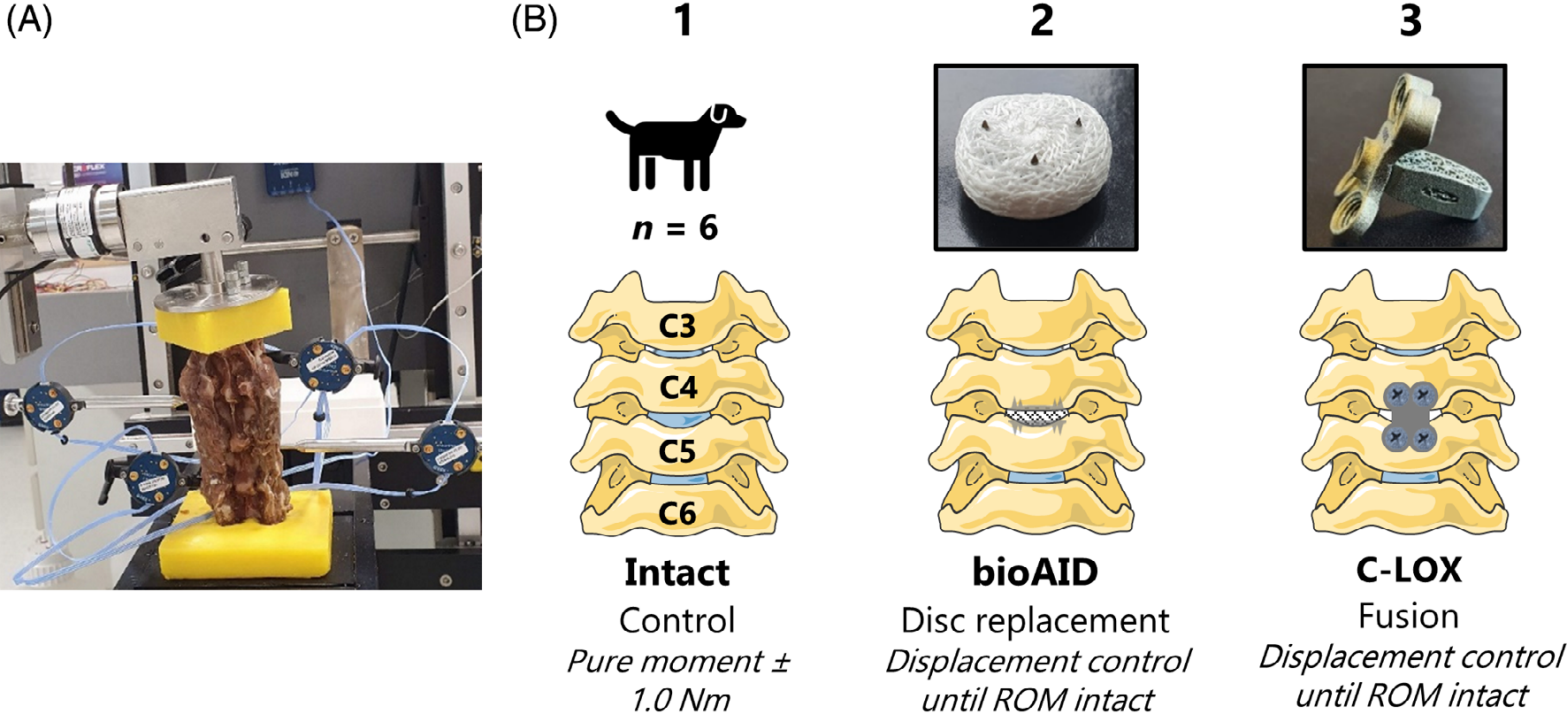
(A) ventral(anterior in humans) view of intact cervical cadaveric canine spine embedded in polymethylmethacrylate resin including insertion of the triplet markers subjected to ±1 Nm pure moment using a 6‐DOF spine tester. (B) schematic representation of the hybrid test protocol in the following three conditions: intact, after replacement of C4‐C5 disc with bioAID, and after C4‐C5 fusion using an anchored cage (C‐LOX). ROM = range of motion. The Figure was partly generated using Servier Medical Art, provided by Servier, licensed under a Creative Commons Attribution 3.0 unported license.

The cadaveric canine specimens (C3‐C6) were subjected to cyclic application (1°/s) of flexion‐extension (FE), lateral bending (LB), and axial rotation (AR) in random order using an electronic 6‐DOF spine testing system capable of applying unconstrained pure moments (Figure 2) (FS21; Applied Test Systems, Buttler, PA, USA).^26^ Each spinal specimen was tested in three conditions: intact, after total disc replacement with the bioAID, and after fusion using an anchored cage (C‐LOX, Rita Leibinger Medical, Muehlheim, Germany) at level C4‐C5. A hybrid protocol was used where the intact spines were first subjected to a pure moment of ±1 Nm for five cycles whereafter the instrumented spines were subjected to the full ROM of the intact condition.^27^ A moment of 1 Nm was selected because it is capable of producing physiologic motions without the risk of damaging spinal structures.^28^ As a result of the low loads applied (±1 Nm) and the flexibility of the cervical spine, the resistances present in the linear actuators on top of the actual weight of the sliding mechanisms could have influenced the natural coupling of motions. It is therefore important to bear in mind that the experimental protocol used in this study cannot fully replicate the in vivo motion behavior of the cervical spine. Motions of the individual vertebrae were obtained with triplet LED‐markers rigidly fixed to each vertebra with custom‐made pins and tracked using an optical registration system (Optotrak Certus, Northern Digital, Waterloo, Ontario, Canada). The data were automatically gathered by the spine tester software (FS21; Applied Test Systems, Buttler, PA, USA) and segmental rotations were calculated using a custom‐written algorithm based on Tait‐Bryan angle sequence (MATLAB R2018b, MathWorks, Natick, MA, USA).^29^ Based on these calculations, data of the fourth cycle was used to determine the segmental ROM in all three degrees of freedom, defined as the difference in rotation at maximum and minimum load. Moment‐Rotation curves were plotted and used to define the neutral zone (NZ), being the region of intervertebral motion around the neutral posture where there is the least resistance. The boundaries of the NZ were defined as the deflection points of compliance in the moment‐rotation curves as described previously.^30^ Data analysis was performed by a customized MATLAB (MathWorks, Natick, MA, USE) script.

### 2.4 Surgical procedure

After testing the intact specimens, the spines were subjected to a near‐complete C4‐C5 discectomy, removing the ventral (anterior in humans) annulus fibrosus and inner layers of the lateral and dorsal (posterior in humans) annulus but leaving the dorsal longitudinal ligament intact. Next, the cartilaginous endplates were scraped using a curette. Before implantation of the bioAID, a custom‐made trial guide was used to drill holes using 1 mm k‐wires into the adjacent vertebral bodies matching the exact locations of the bioAID endplate pins. After testing the spinal specimens with the bioAID, the implant was removed. Next, a smooth trial guide was used to assess the appropriate size of the anchored cage (C‐LOX, Rita Leibinger Medical, Muehlheim, Germany). The appropriate size cage with spikes was then attached to an insertion tool and hammered into the correct position within the disc space and fixated with four titanium locking screws before being tested with the spine tester.

### 2.5 Intradiscal pressure

A pressure measuring sensor (type CTN0 4F HP, Gaeltec Devices Ltd, Dunvegan, Isle of Skye, Scotland, UK) was positioned in the C3‐C4 IVD to assess changes in IDP of intact compared to treated spines. A 1.2 mm‐diameter needle was manually pushed through the ventral (anterior in humans) annulus fibrosus into the center of the nucleus pulposus. The needle was removed, and the pressure transducer needle was inserted into the created channel. During the loading cycles, the voltage outputs of the pressure sensor were recorded continuously using a universal amplifier (MPAQ, IDEE/Maastricht Instruments, Maastricht, The Netherlands). Peak pressures of the fourth loading cycle were reported.

### 2.6 Statistical analysis

ROM and NZ data were normalized to the intact condition to account for differences between specimens. Mean values and standard deviation were calculated for each parameter. Comparisons between experimental groups of ROMs, NZ, and IDP data were determined by repeated measures ANOVA (with Geisser–Greenhouse correction), or mixed effect analysis when there were missing values, followed by Tukey's honest post hoc analysis(GraphPad Prism version 8.0.2 for Windows, San Diego, California USA). In all cases, *p* < 0.05 was defined as a statistically significant difference.

## 3 RESULTS

### 3.1 Segmental ROM

**FIGURE 3:**
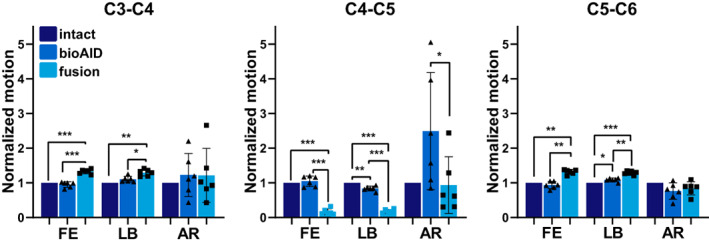
Normalized segmental range of motion ± standard deviation for spinal levels between C3‐C6 in flexion/extension (FE), lateral bending (LB), and axial rotation (AR) for intact C4‐C5 disc, after replacement with bioAID, and after C4‐C5 fusion in intact specimens, with bioAID and fusion at C4‐C5. (Repeated measures ANOVA, Tukey post hoc: *p < 0.05; **p < 0.01; ***p < 0.001).

The bioAID provided similar ROM compared with the intact segment during FE (105% ± 14% of intact) at the treated level (Figure 3 and Table 2). In AR, an increase in mean ROM was observed at C4‐C5, showing 249% ± 154% of the intact ROM after disc replacement with the bioAID. Also at the adjacent levels, the ROM was preserved and statistically similar to intact after replacement with the bioAID for both FE (C3‐C4, 95% ± 8% of intact; C5‐C6, 94% ± 9% of intact) and AR (C3‐C4, 122% ± 57% of intact; C5‐C6, 76% ± 22% of intact). During LB, 84% ± 6% of the intact ROM was found at level C4‐C5 for the bioAID, being significantly lower than the intact condition. The reduced motion at the treated level led to increased motion at the adjacent levels (C3‐C4, 111% ± 9% of intact; C5‐C6, 108% ± 5% of intact).

**TABLE 2:** Normalized mean range of motion ± standard deviation (SD), and mean moment ± SD during spine testing in three directions for intact C4‐C5 disc, after replacement with bioAID at C4‐C5 and after C4‐C5 fusion. Significantly different compared with intact measured with repeated ANOVA, Tukey post hoc.

	Flexion/extension			Lateral Bending			Axial Rotation		
	Intact	BioAID	Fusion	Intact	BioAID	Fusion	Intact	BioAID	Fusion
Mean moment	1.05	0.91	1.80*	1.03	1.23	2.26**	1.13	0.80*	1.31
SD	0.03	0.45	0.56	0.06	0.33	0.59	0.06	0.70	0.29
Normalized ROM	‐	1.05	0.17***	‐	0.84**	0.18***	‐	2.49	0.93
SD	‐	0.14	0.08	‐	0.06	0.05	‐	1.54	0.75

* *p* < 0.05.

** *p* < 0.01.

*** *p* < 0.001.

In contrast to replacing the IVD with the bioAID, fusing the spine at level C4‐C5 led to a significant loss of motion in FE (17% ± 8% of intact) and LB (18% ± 5% of intact).In direct relation to this loss of motion at the treated level, levels adjacent to fused segments showed a significantly increased motion in FE (C3‐C4, 133% ± 6% of intact; C5‐C6, 132% ± 6% of intact) and LB (C3‐C4, 111% ± 9%, and, C5‐C6 (129% ± 5% of intact). However, in AR, the ROM at C4‐C5 remained close to the intact condition (93% ± 75% of intact). As a result, also at the adjacent level, the ROM in AR remained statistically equivalent to intact (C3‐C4, 122% ± 9% of intact; C5‐C6, 84% ± 17% of intact).

Table 2 shows the amount of torque (Nm) required to achieve the intact ROM after disc replacement with the bioAID and after fusion. After disc replacement with the bioAID, the moment data were close to the intact moment in FE and LB, but approximately 30% less in AR.

The fused specimens required the highest torque to achieve the intact ROM in all directions. This was only significant in FE and LB where the required moment was almost double the moment seen in the intact condition.

### 3.2 Neutral zone

**FIGURE 4:**
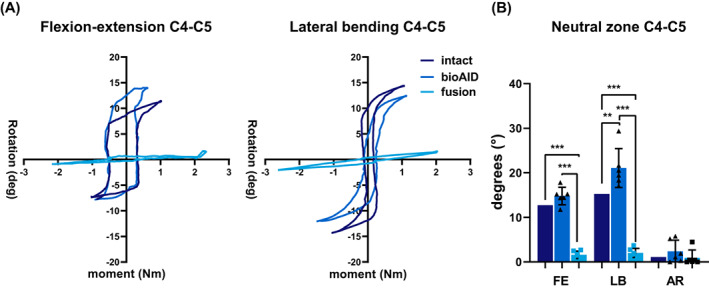
Representative moment‐rotation curves (A) and mean normalized neutral zone ± standard deviation (B) for intact C4‐C5 disc, after replacement with bioAID at C4‐C5 and after C4‐C5 fusion.(Repeated measures ANOVA, Tukey post hoc:**p < 0.01; ***p < 0.001).

Based on the moment‐rotation graphs (Figure 4A), the bioAID exhibited nonlinear behavior with a neutral and elastic zone comparable to what was seen in the intact condition at the treated level for both LB and FE. When quantifying the normalized NZ, results showed that the bioAID indeed had a NZ close to intact in FE (Figure 4B). However, a significantly smaller NZ was observed in LB for the bioAID (Figure 4B). For the fused segments, no NZ could be identified for FE and LB. A small NZ in AR was detected for all three conditions (Figure 4B).

### 3.3 Intradiscal pressure

**FIGURE 5:**
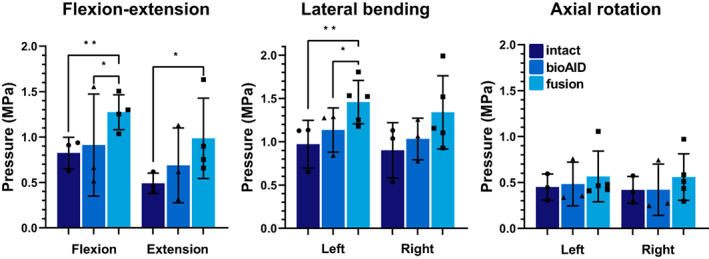
Intradiscal pressure (MPa, mean ± standard deviation) at C3‐C4 for intact, after replacement of C4‐C5 with bioAID and after C4‐C5 fusion. (Mixed effect analysis, Tukey post hoc: *p < 0.05, **p < 0.01).

After implantation of the bioAID, the peak IDP was similar at the adjacent cranial level compared with intact in all directions (Figure 5). An increase in the mean IDP was observed for all three DOF at the adjacent cranial level of the fused specimens compared with the intact spines, although only significant in FE and LB.

## 4 DISCUSSION

To assess the initial biomechanical effect of the bioAID on the kinematic behavior of the spine an ex vivo biomechanical study in 6‐DOF was performed. The current study found that the bioAID can preserve motion at treated and adjacent levels and shows similar nonlinear behavior including a NZ as seen for the intact condition, indicating its potential to restore physiological kinematics and similar ROM allowed by the spinal segments. Unlike fusion, by preserving this kinematic signature and adjacent intradiscal pressures, the bioAID might reduce overloading of the surrounding structures, thereby potentially reducing the risk of adjacent segment disease.

The most important and most reported parameters to assess the biomechanical similarities between intact and treated specimens are the ROM and neutral zone.^31^ Within the context of disc replacement, the NZ is often seen as a clinically relevant measure of the quality of motion, giving information about the region of intervertebral motion where there is the least resistance. It is hypothesized that alterations in the sigmoid curve, also called the kinematic signature, can result in altered stresses on the spinal musculature and ligaments that stabilize the spinal segment.^32^ The similarities in the sigmoid curve observed seem to indicate that the bioAID allows for similar semi‐constrained motion as the native disc and thus can better replicate the kinematics of the intact condition compared to spinal fusion.

Besides the similarities in FE and the sigmoid shape of the moment‐rotation curves, significant differences in ROM during LB and AR were observed. Similar to Patwardhan (2012) and Snyder (2007), the decrease in LB after CDR with the bioAID could be attributed to the fact that a small part of the lateral and dorsal borders of the annulus fibrosus (or equivalent anatomical locations) was preserved to reduce risk of migration and preserve additional stability.^21^, ^33^

However, the most distinct difference in ex vivo motion between the bioAID and the intact condition was during AR, where especially two specimens show a much higher ROM compared to the other results. This difference could be explained by the lack of initial fixation since these two specimens were instrumented with previously implanted bioAIDs due to the limited availability of bioAIDs, resulting in flattened pins due to the retraction procedure. This instability mainly affected AR since this motion results in a shearing force. Within this context, the fibers of the jacket play an important role in resisting shearing motion, similar to Sharpey's fibers of the natural annulus.^34^ To mimic the kinematic behavior of a natural IVD, shearing needs to be transferred through the jacket, which cannot be achieved without proper interconnection between vertebrae and fibers of the jacket. This can also explain why even for the samples with intact initial fixation, a slight increase in AR for the bioAID compared to intact was observed. Previous finite element modeling research on the bioAID also found that, especially for AR, bone in growth over the whole cranial and caudal surface of the implant is required to mimic the motion of the intact condition.^24^ In vivo, osseointegration between the jacket's fibers and the vertebrae is lacking immediately post‐surgery. As bone ingrowth takes time, the current fixation system is probably still sufficient as initial fixation, providing similar motion characteristics in FE and LB.

After fusing the spines at level C4‐C5, the ROM was redistributed over the three segments, similar to what has been reported in other studies that utilized a hybrid protocol.^35^, ^36^, ^37^, ^38^ It is hypothesized that the altered motion pattern at the treated level often leads to a compensatory mechanism at the adjacent levels, which can increase the risk of adjacent segment disease in the long term.^8^, ^35^ Surprisingly, in AR, no significant reduction in ROM was observed. Other studies have also reported the least difference in AR after fusing the segment,^39^, ^40^ but contradictive results have also been reported.,^36^, ^37^, ^41^, ^42^, ^43^, ^44^ A possible explanation for the reported discrepancies could be the use of different cage designs and the lack of bone ingrowth.

By using the hybrid protocol, the measurements performed on the instrumented spines can result in different peak torques acting on the specimens for reaching the same global ROM as the intact condition. Comparing the ROM alone is therefore not sufficient to verify similarities between instrumented and intact spines. Based on the results, the fused specimens needed much higher moments to reach the same global ROM, indicating that an increased force is required to preserve cervical physiological ROM after fusion similar to what has been observed in other in vivo and in vitro research.^8^, ^35^, ^36^, ^38^, ^45^ This hybrid protocol was chosen based on the hypothesis of Panjabi et al. (2007), stating that a modification, in this case, a disc replacement with the bioAID or fusion, will result in a compensatory mechanism of the adjacent levels resulting in a redistribution of the loads to reach the intact condition.^27^ Many other studies utilize the flexibility protocol, in which a constant pure moment is applied in all three conditions while measuring the resulting ROM.^39^, ^40^, ^42^, ^43^, ^46^, ^47^ However, the flexibility method is unable to evaluate the effect on the adjacent levels since it applies an equal moment at all spinal levels and thus is less suitable to identify if there are alterations in the overall kinematic behavior of the spine after treatment.

Lastly, the IDP provides more insight into the adjacent level kinematics and the redistribution of biomechanical stresses acting on the spine after treatment. The preserved IDP after disc replacement with the bioAID indicates there is a similar distribution of biomechanical stresses as in an intact spine. Previous studies have also shown that preserving motion has a positive effect on preserving IDP at the adjacent levels.,^35^, ^48^, ^49^, ^50^, ^51^ After fusing the segment, the loss of motion at the fused level led to elevated IDPs at the adjacent level compared with intact in all directions. These observations again demonstrate that adjacent segments are compensating for the loss of motion and that there is an altered loading pattern in the spine, as also seen in ROM, coupled motion, and NZ data. Other studies also observed that loss of motion at the treated level leads to compensation mechanisms at the adjacent level, such as elevated IDPs.^35^, ^52^

There are several study limitations and considerations to the interpretation of the current results which also make a direct comparison with other studies difficult due to differences in specimen origin, specimen quality, testing protocol, surgical procedure, and testing apparatus.

First of all, the bioAIDs were implanted in a swollen condition giving the bioAID a final height of approximately 6 mm. This could have led to over distraction of the disc space imposing increased tension on soft spinal structures that potentially limit ROM. This was done to best replicate the motion behavior after reaching swelling equilibrium in vivo since unconstrained swelling of the hydrogel can take up to 6 days in a physiologic salt solution.^53^ Ultimately, the bioAID will be implanted unswollen to avoid this and allow swelling until equilibrium under physiological loading. Moreover, differences in height between the bioAID and fusion cages could have affected soft‐tissue tensioning between the two conditions, potentially influencing the kinematics.

This experiment only used one size of the implant while, in general, the implant is adjusted to the dimensions of the patient. As a result, for some spinal specimens, the implant was slightly too big, potentially hampering the ROM. This could explain the variations observed, but it did not affect the overall trend seen.

It must also be mentioned that in the current study the use of a follower load to replicate the muscle forces that act on the cervical spine was omitted. In general, including a follower load leads to stiffening of the IVD and thus often results in decreased ROM and NZ and increased IDPs, especially in FE.^50^, ^52^, ^54^ This effect might be even more prominent for the bioAID, since this design contains a compressible core, which is seen as one of the advantages, giving the device its shock absorption capability. However, it is also speculated that incorporating a follower load in an ex vivo setting might result in applying unphysiological forces, especially during rotation.^55^ Although a follower load was not incorporated in this research, current results still illustrate that the bioAID design allowed motion based on deformation and was able to mimic both the ROM in FE, NZ, and IDP as seen in the intact condition.

The serial nature of this repeated‐measures experiment could have introduced iatrogenic changes during the intact and/or bioAID conditions influencing the results of the fusion condition. Although this is unlikely under such low loads, future work should be carried out to confirm this.

Lastly, this study cannot fully elucidate its benefits compared with first‐generation ball‐and‐socket designs. The rationale of the bioAID design is that by mimicking the structure of the natural IVD, it can better replicate the kinematics of a native IVD compared with first‐generation ball‐and‐socket designs. Based on the data of this study, it can be suggested that the bioAID can restore motion and allow for nonlinear behavior similar to an intact spine at both the adjacent and treated level. Despite these promising results, actual improvements in this design compared with ball‐and‐socket first‐generation devices cannot be deducted from this study. To assess differences between these designs, clinical trials with long‐term follow‐up data are necessary. Both design categories can maintain motion, but the biomimetic design aims to reduce compensatory mechanisms at the adjacent levels with the hypothesis that this will lead to a reduced risk of adjacent segment disease in the long term.

## 5 CONCLUSION

In spite of its limitations, the results obtained in this research illustrate that the bioAID may preserve the adjacent level IDPs and segmental kinematics close to the intact condition. These findings support the hypothesis that CDR using the novel bioAID can be a promising alternative treatment for replacing severely degenerated IVDs. By maintaining normal kinematics and stresses at the treated and adjacent levels, the bioAID might minimize the risk of adjacent segment disease. However, further preclinical work based on in vivo evaluation in a large animal model is needed prior to making valid conclusions regarding its safety and efficacy in comparison to other current treatment options.

## AUTHOR CONTRIBUTIONS

Celien A.M. Jacobs, Remco J.P. Doodkorte, Keita Ito conceived and designed the study. Celien A.M. Jacobs, Remco J.P. Doodkorte, S. Amir Kamali, Björn P. Meij performed the experimental work. Celien A.M. Jacobs, with help of Remco J.P. Doodkorte, obtained and analyzed the data presented in this publication. Abdelrahman M. Abdelgawad, Samaneh Ghazanfari, Stefan Jockenhoevel, provided input on the design of the fiber jacket. Critical revising of the article was done by all co‐authors. Approval of the submitted version was given by all authors.

## CONFLICT OF INTEREST STATEMENT

Keita Ito is an Editorial Board member of JOR Spine and a co‐author of this article. To minimize bias, they were excluded from all editorial decision‐making related to the acceptance of this article for publication. [Correction added on 22 June 2023, after first online publication: Conflict of Interest statement was revised]
